# NiTi instruments in endodontics: contemporary concepts, principles, and practical guidance

**DOI:** 10.1590/1807-3107bor-2026.vol40.027

**Published:** 2026-05-11

**Authors:** Emmanuel João Nogueira Leal da SILVA, Jorge Nuno Rosário MARTINS, Marco Aurélio VERSIANI

**Affiliations:** (a) Universidade do Estado do Rio de Janeiro – UERJ, School of Dentistry, Department of Endodontics, Rio de Janeiro, RJ, Brazil.; (b) Universidade de Lisboa – UL, School of Dentistry, Department of Endodontics, Lisboa, Portugal.; (c) Polícia Militar de Minas Gerais – PMMG, Dental Speciality Center, Belo Horizonte, MG, Brazil.

**Keywords:** Mechanical Tests, Metallurgy, Root Canal Preparation

## Abstract

This review synthesizes current laboratory and clinical evidence on nickel-titanium (NiTi) endodontic instruments and translates it into guidance for clinical practice, addressing inconsistencies in the translation of research findings and a lack of standardization. Evidence on the evolution, metallurgy, heat treatments (e.g., M-Wire, Gold, and Blue), geometric design, mechanical properties, and clinical performance of contemporary NiTi systems were synthesized, with emphasis on shaping outcomes, including canal transportation, volumetric changes, unprepared canal walls, and remaining dentin thickness. Modern NiTi instruments, particularly those with advanced heat treatments, provide superior flexibility and cyclic fatigue resistance compared with conventional alloys, enabling safer and more predictable canal preparation. Continuous rotary systems often achieve better canal centering and less transportation than reciprocating systems; however, both leave a substantial portion of canal walls untouched, highlighting the essential role of chemical debridement. Long-term clinical success depends primarily on biological factors and careful technique rather than on the specific instrument system. Therefore, NiTi instruments represent the standard of care, offering anatomically respectful canal shaping. Clinicians should integrate knowledge of alloy properties, design features, and failure mechanisms with conservative, load-managing techniques to maximize safety and efficiency.

## Introduction

Root canal shaping is a fundamental step in endodontic therapy because it defines the three-dimensional space required for effective irrigation, disinfection, and obturation of the root canal system. Nickel-titanium (NiTi) instruments have enhanced shaping procedures by combining superelasticity and shape memory with optimized design and controlled motion. Compared with stainless-steel files, NiTi instruments better preserve the original canal path and reduce canal transportation.^1^ The selected preparation strategy directly affects clinical outcomes because NiTi instruments reduce the risk of iatrogenic errors, such as ledges, zips, and strip perforations. They also improve centering and maintain a consistent taper, which facilitates irrigant penetration into complex canal anatomy. Taken together, these advantages support more effective chemomechanical cleaning and create conditions that favor periapical healing.^2^

The performance of NiTi systems depends on alloy composition and heat treatment, taper and core mass, cross-sectional design, and kinematics. These features determine flexibility, torsional strength, resistance to cyclic fatigue, cutting efficiency, canal cleanliness, dentin preservation, and the risk of instrument separation.^3,4^ Together, they influence procedural safety and clinical outcomes, such as periapical repair, symptom relief, and long-term tooth retention.^5^ These parameters are clinically relevant because they affect how effectively clinicians disinfect the canal system while preserving tooth structure and preventing procedural errors. Excessive dentin removal weakens roots and increases fracture risk, whereas insufficient shaping restricts irrigant exchange and reduces bacterial control. Therefore, instrument design and kinematics exert direct biological and mechanical effects on treatment outcomes.

Despite extensive laboratory and clinical research, the translation of research findings into clinical practice remains inconsistent.^4^ Manufacturers apply different heat treatment protocols and report phase transformation temperatures inconsistently, which limits direct comparison among systems. Mechanical testing also lacks standardization, with wide variation in temperature control, irrigant composition, and loading conditions. In addition, key mechanical concepts remain poorly differentiated in publications and training, including bending versus buckling, axial versus lateral cutting, and torsional overload versus cyclic fatigue failure. These gaps affect clinical decision-making. Clinicians must define safe reuse and discard criteria, adjust motor torque and speed, select motion strategies for complex anatomies, and verify product authenticity in a market affected by replicas. The growing use of cone-beam computed tomography and navigation or artificial intelligence-based software further influences sequence planning and risk assessment. However, standardized clinical protocols for integrating these tools remain limited, contributing to outcome variability.

The objective of this paper is to provide a comprehensive synthesis of current laboratory and clinical evidence on NiTi endodontic instruments, highlighting how alloy properties, instrument design, and procedural techniques influence shaping efficiency, safety, and clinical outcomes, and to offer practical guidance for optimizing their use in routine root canal treatment.

## Historical background and evolution

For most of the twentieth century, root canal shaping relied on stainless-steel hand instruments such as K-files and reamers. Clinicians applied step-back,^6^ crown-down,^7^ watch-winding,^8^ and later balanced-force^9^ techniques to shape canals. Because stainless steel has a high elastic modulus, these instruments tended to straighten within curved canals and concentrate stress at points of curvature. Consequently, successful treatment depended on careful precurving and precise tactile control to follow canal anatomy and limit procedural errors, such as ledges, zips, elbows, apical blockage, canal transportation, and strip perforations. Gates-Glidden drills helped enlarge the coronal third but increased the risk of strip perforation when overused. Altogether, these limitations made shaping highly technique-sensitive and strongly dependent on canal anatomy.^1^

In the early 1960s, researchers at the United States Naval Ordnance Laboratory, led by William J. Buehler, developed a near-equiatomic NiTi alloy known as Nitinol. Their objective was to produce materials with high fatigue resistance and thermal stability for military and aerospace applications. The alloy exhibited shape memory, defined as the ability to return to a preset shape after heating, and superelasticity, defined as the capacity to undergo large reversible strains under near-constant stress. These characteristics established NiTi as a distinct metallurgical class rather than a variant of stainless steel^10^ and stimulated interest in medical and dental applications. Civjan et al.^11^ proposed the use of NiTi in dentistry because of its shape memory and low elastic modulus, which made it suitable for curved and anatomically complex spaces. Orthodontics adopted the alloy early, with Andreasen and Morrow^12^ reporting laboratory and clinical studies showing that Nitinol wires provided improved control of tooth movement because of their low elastic modulus and near-constant force delivery. Taken together, these findings demonstrated that NiTi alloy could function reliably in the oral environment.

A major advance in endodontics occurred when Walia et al.^13^ manufactured prototype K-files from orthodontic NiTi wire. They demonstrated that these instruments had two- to three-fold greater bending flexibility and higher resistance to torsional fracture than stainless-steel files with similar geometry. This improvement reduced the mechanical factors that contribute to ledges, canal transportation, and other procedural errors in curved canals. Consequently, the study positioned NiTi as a practical material for endodontic instruments and encouraged targeted product development.

Despite these advantages, NiTi hand files were not widely adopted in clinical practice and did not lead to major improvements in canal shaping. This was primarily because they still required manual use, which is strongly influenced by operator skill. In addition, hand-driven motion was slow and irregular, limiting effective use of the superelastic properties of NiTi. Consequently, the potential benefits of NiTi were largely unrealized until the alloy was used with continuous rotary motion. Rotary movement provided more consistent and controlled loading, enabling more stable and predictable enlargement of the root canal.

**Figure 1:**
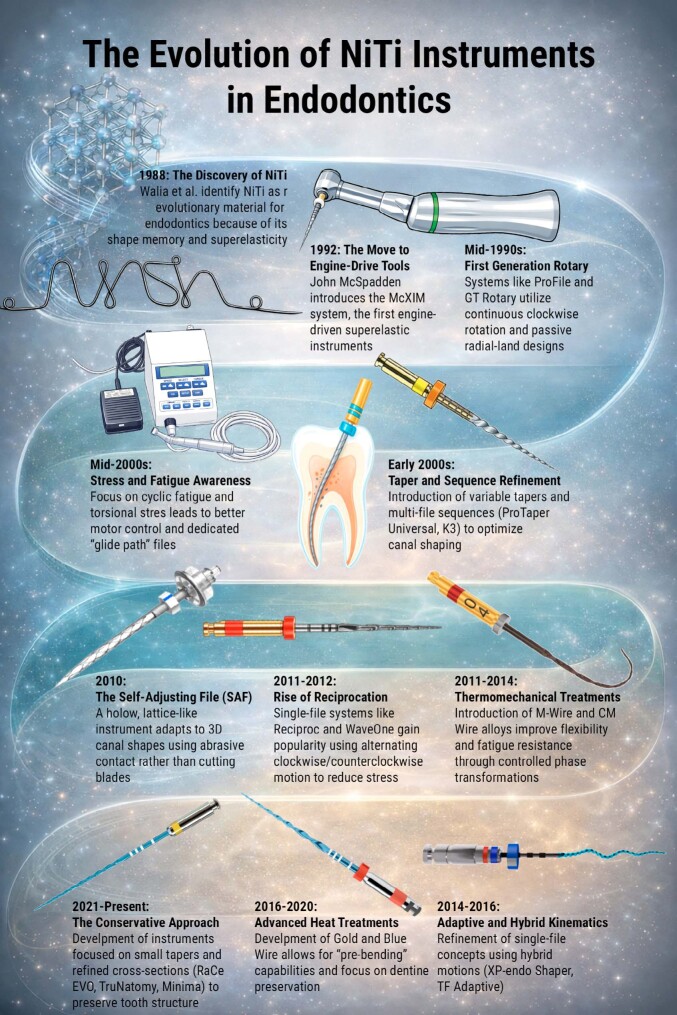
Evolution of NiTi instruments in endodontics. Schematic timeline showing key technological milestones, from the introduction of NiTi alloys and engine-driven rotary systems to reciprocating kinematics, thermomechanically treated alloys, and contemporary conservative shaping concepts aimed at improving flexibility, fatigue resistance, and dentin preservation.

In 1994, NT Co. launched the first set of NiTi rotary instruments with multiple nonstandard tapers, known as the McXIM Series. This series included six files with taper sizes ranging from the conventional 0.02 to 0.05 and was designed to reduce stress by limiting instrument contact during sequential rotary enlargement. Following early success and the clear benefits reported, NiTi rotary instruments quickly gained widespread clinical adoption^14^ (Figure 1). Thereafter, manufacturers began producing NiTi instruments with standardized tapers and geometries; systems such as ProFile (Dentsply Maillefer), Quantec (Analytic/SybronEndo), and GT files (Dentsply Tulsa) marked the consolidation of the rotary era. The introduction of torque-controlled motors further enhanced operational safety and consistency during canal preparation, establishing rotary NiTi shaping as a clinical standard and laying the technical groundwork for later innovations, including reciprocating kinematics, single-file systems, and proprietary heat-treated alloys.

In light of this rapid technological progression, root canal preparation instruments have been classified according to multiple criteria, including date of introduction, geometric configuration (active portion, cross-sectional design, tip, taper, helical angle, etc.), NiTi alloy and manufacturing process, and activation kinematics. However, a more comprehensive and clinically meaningful approach is to classify these instruments into generations based on the progressive evolution of their design features and underlying technologies (Table).

The table presents selected, well-established systems and is not intended to provide a comprehensive list of all instruments currently available or previously introduced.

### Metallurgy and heat treatments

NiTi alloys have transformed endodontic practice by providing flexibility, shape memory, and superelasticity not achievable with stainless-steel instruments. These functional properties derive from the near-equiatomic composition of the alloy, typically about 56 wt% nickel and 44 wt% titanium, which enables temperature- and stress-driven phase transformations.^15,16^ At higher temperatures, the alloy remains in the austenite phase, characterized by a cubic B2 lattice and higher stiffness. As temperature decreases or with applied stress, the structure changes to martensite, which has a monoclinic B19’ lattice and shows greater capacity for plastic deformation. Under specific thermal and mechanical conditions, an intermediate R-phase with a rhombohedral lattice can form between austenite and martensite.^17^

These phase transformations explain the clinical behavior of NiTi instruments. When the alloy deforms in the martensitic state at lower temperatures, it recovers its original shape upon heating above the austenite-finish temperature, producing the shape memory effect. When instruments operate at temperatures above the austenite-finish temperature, applied stress alone can trigger a reversible transformation from austenite to stress-induced martensite and back, resulting in superelasticity^15,16^. This stress-driven mechanism allows NiTi instruments to negotiate and enlarge complex root canal anatomies, such as severely curved, narrow, or multiplanar canals, with greater efficiency and safety than stainless-steel instruments, which tend to straighten and plastically deform under similar conditions. Consequently, NiTi instruments can prepare anatomies that would be difficult or even impractical to manage using conventional stainless-steel manual instrumentation.

Most dental NiTi alloys maintain a near-equiatomic composition; however, small compositional variations markedly influence transformation behavior.^18,19^ Minor changes in nickel or titanium content can shift the martensite-start, martensite-finish, austenite-start, and austenite-finish temperatures by several degrees.^15,17^ Nickel-rich alloys lower transformation temperatures and stabilize the austenite phase at clinical temperatures, whereas titanium-rich formulations raise transformation temperatures and favor the martensitic phase under the same conditions.^15^ In addition, trace alloying elements, such as cobalt or iron, and the formation of secondary phases, such as Ti_2_Ni, can further modify transformation temperatures and alter mechanical behavior.^20^ This high sensitivity to composition highlights the need for strict control of alloy chemistry and standardized heat treatment to ensure predictable clinical performance.

### Conventional NiTi wires and their limitations

The first rotary instruments were made from conventional superelastic NiTi wire and operated mainly in the austenite phase at clinical temperatures (Table 1). These instruments performed better than stainless-steel instruments, but they often fractured suddenly because of cyclic fatigue or excessive torsional stress.^21,22^ Microstructural studies showed extensive cold working and residual stresses from manufacturing, which disrupted phase transformation and reduced fatigue resistance.^21^ Because the austenite-finish temperature of conventional NiTi remained well below clinical temperatures, the alloy stayed fully austenitic during use. Consequently, the instruments remained relatively stiff, exhibited strong elastic springback, and tended to straighten curved canals. This behavior increased the risk of canal transportation and ledge formation.^1,16,17^ These mechanical and clinical limitations led to the development of advanced metallurgical approaches, particularly controlled heat treatments, to shift transformation temperatures, increase martensitic content at operating temperatures, improve flexibility, and reduce fracture risk.

**Table:** Evolution of nickel–titanium (NiTi) rotary and reciprocating endodontic instruments by generation.

Generation	Systems	Characteristics
1st generation	McXim File (NT Company), GT system (Dentsply), LightSpeed (LightSpeed Technology), Pow-R (Moyco), ProFile (Dentsply), ProFile Series 29 (Dentsply), Quantec (Tycom), Quantec 2000 (Tycom), Rapid Body Shaper (Moyco)	Constant taper designs with passive radial lands and neutral or negative cutting angles, enabling mechanized canal preparation but requiring complex multi-instrument protocols and showing high rates of deformation and fracture due to early manufacturing limitations and limited clinical experience.
2nd generation	BioRaCe (FKG), EndoSequence (Brasseler), K3 (Sybron), Mtwo (VDW), ProTaper Universal (Dentsply)	Surface-treated and modified geometries with active cutting edges and no radial lands, resulting in greater cutting efficiency, fewer instruments per sequence, and generally lower fracture rates than first-generation systems, although instrument separation remained a relevant clinical concern requiring glide path preparation and careful use.
3rd generation	HyFlex CM (Coltène), K3XF (Sybron Endo), ProFile GT Series X (Dentsply), ProFile Vortex (Dentsply), ProFile Vortex Blue (Dentsply), ProTaper Gold (Dentsply), Twisted File (Sybron), Typhoon (Clinician’s Choice)	Thermomechanical heat treatment of the alloy (Superelastic NiTi), resulting in enhanced fatigue resistance, improved safety and shaping ability, particularly in curved canals, and reduced instrument sequences compared with earlier generations, while maintaining similar clinical precautions.
4th generation	Reciproc (VDW), Reciproc Blue (VDW), WaveOne (Dentsply), WaveOne Gold (Dentsply), Twisted-File Adaptive (Sybron Endo), Self-Adjusting File (Redent Nova)	Introduction of reciprocating kinematics, in which asymmetric oscillatory motion replaces continuous rotation to reduce torsional stress, enhance fatigue resistance, and extend instrument lifespan while maintaining the metallurgical advances of earlier generations.
5th generation	OneShape (MicroMega), ProTaper Next (Dentsply), Revo-S (MicroMega), Rotary Files (VDW), TruNatomy (Dentsply), TruShape (Dentsply), XP EndoShaper (FKG)	Advanced metallurgical treatments and off-center, asymmetric designs that produce eccentric rotation and, in some systems, temperature-dependent expansion, aiming to enhance shaping efficiency and canal wall contact despite limited evidence of superior shaping outcomes over conventional designs.

### Heat treatment of NiTi alloys

Heat treatment refers to thermal and mechanical processes applied to NiTi alloys to change microstructure, relieve internal stress, and adjust transformation temperatures. These changes shift the balance between martensite, austenite, and R-phase at endodontic service temperatures, thereby increasing flexibility and fatigue resistance.^17,21^ Manufacturers combine controlled heating, cooling rates, and mechanical processing to obtain predictable phase distributions and mechanical behavior. Over the last two decades, several categories of heat-treated NiTi wires have reached the market (Table 1).

M-Wire (Dentsply Sirona) was one of the first proprietary alloys and results from a pre-manufacturing thermal treatment applied during wire manufacture. Instruments made from this alloy, such as WaveOne (Dentsply Sirona), Reciproc (VDW), ProTaper Next (Dentsply Sirona), and ProTaper Ultimate Slider (Dentsply Sirona), showed higher cyclic fatigue resistance than conventional NiTi while maintaining adequate stiffness for cutting efficiency.^17^

R-Phase wire describes heat-treated NiTi used in some instruments, although the wire does not always exhibit a true R-phase crystal structure. Manufacturers may apply heat treatment before twisting, as in Twisted Files (SybronEndo), or after grinding, as in K3XF (SybronEndo). This processing lowers shear modulus and reduces surface residual stress, which increases flexibility and cyclic fatigue resistance, even though the alloy remains predominantly austenitic at body temperature.^17^

Controlled-Memory wire, first used in HyFlex CM (Coltene-Whaledent) and later in other systems, has an austenite-finish temperature above body temperature. Instruments therefore remain partly martensitic at intracanal temperatures. This phase state explains their high flexibility, limited superelastic response, and low springback, which make them suitable for canals with abrupt curvature.^17,22^

Gold wire is used in WaveOne Gold (Dentsply Sirona), ProTaper Gold (Dentsply Sirona), and some ProTaper Ultimate (Dentsply Sirona) instruments. Postmanufacturing heat treatment produces a yellow oxide surface layer, which increases the proportion of martensite and the R-phase at service temperatures, improving flexibility and cyclic fatigue resistance compared with M-Wire and conventional NiTi.^17,23^

Blue wire is used in Reciproc Blue (VDW), ProFile Vortex Blue (Dentsply Tulsa), and ProTaper Ultimate FX and FXL instruments (Dentsply Sirona). A postmanufacturing heat treatment creates a blue oxide surface layer. Phase analysis shows a martensitic structure at room temperature and a more austenitic structure at body temperature, which increases ductility and cyclic fatigue resistance at room temperature. Because this alloy exhibits a higher austenite fraction at body temperature, it tends to be less flexible than Gold heat-treated alloys.^24^

Surface color alone does not define the metallurgical properties of NiTi instruments. The yellow or blue appearance simply reflects a superficial oxide layer and does not correspond to a standardized heat treatment. Similarly, the multicolored pattern observed in RCS Rainbow (Ramo Medical) NiTi instruments results from the optical interference effects of thin-film surface coatings, such as diamond-like carbon (DLC), rather than from differences in alloy composition, phase transformation temperatures, or mechanical behavior.^25^ As demonstrated for the RCS Rainbow (Ramo Medical) system, these colors are an aesthetic consequence of the coating process and do not indicate any specific mechanical characteristic or performance advantage. Each manufacturer applies distinct heating, cooling, or surface-modification protocols, producing different phase compositions and microstructures. Consequently, instruments with similar or multicolored surfaces may exhibit markedly different flexibility, strength, or fatigue resistance. Clinicians should therefore avoid assuming material equivalence or enhanced performance based solely on instrument color.

### Phase transformation temperatures and differential scanning calorimetry (DSC) testing

The mechanical behavior of NiTi instruments during clinical use is largely determined by their phase transformation temperatures, including martensite start (Ms), martensite finish (Mf), austenite start (As), and austenite finish (Af). In practical terms, these parameters indicate whether an instrument behaves predominantly as a more flexible material (martensitic), a stiffer and more spring-like material (austenitic), or a combination of both at a given temperature. This distinction is clinically relevant because martensitic instruments are generally more flexible and can adapt more easily to curved canals, reducing the risk of canal transportation. In contrast, austenitic instruments exhibit greater elastic recovery and a higher restoring force, which can be advantageous in situations requiring stronger cutting and shape recovery, such as retreatment procedures or instrumentation of narrow and calcified canals, in which effective penetration and maintenance of the instrument’s original shape are required. Consequently, knowledge of transformation temperatures helps predict flexibility, shape recovery, and fatigue resistance under intracanal conditions.

**Figure 2:**
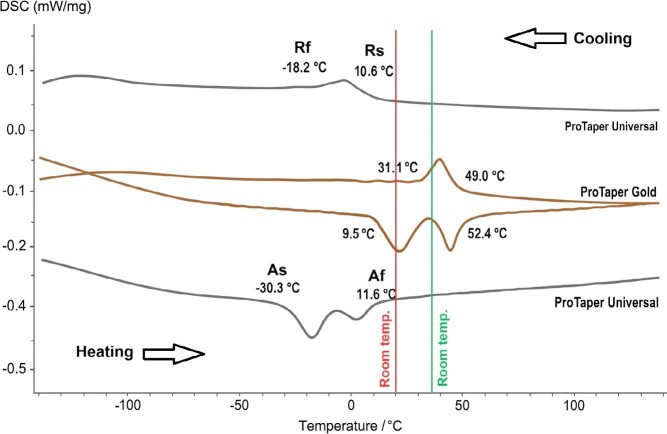
Differential scanning calorimetry (DSC) chart of ProTaper Universal (Dentsply Tulsa) and ProTaper Gold (Dentsply Sirona) NiTi instruments. The Rs and Rf values for ProTaper Universal (Dentsply Tulsa) ranged from 10.6°C to −18.2°C, conferring an austenitic character at endodontic service temperatures (20°C to 36°C). In contrast, ProTaper Gold (Dentsply Sirona) showed Rs and Rf values ranging from 49.0°C to 31.1°C, indicating predominantly martensitic characteristics within the same temperature range. Although geometrically similar, these instruments are expected to exhibit distinct mechanical behaviors due to their different crystallographic arrangements. The DSC data were obtained from Martins et al.^23^ The upper curves represent cooling and should be read from right to left, whereas the lower curves represent heating and should be read from left to right.

Differential scanning calorimetry (DSC) is the standard method used to measure these transformation points. DSC measures the heat absorbed or released by the alloy during controlled heating and cooling, allowing precise identification of the temperatures at which phase changes occur.^20^ DSC also identifies R-phase start (Rs) and R-phase finish (Rf) temperatures, which further explain phase stability at endodontic service temperatures. Studies using DSC have shown that Gold wire instruments often present Rs values during cooling close to 50°C (Figure 2), indicating that they remain partly martensitic or in a mixed phase at body temperature and therefore exhibit higher flexibility and reduced superelasticity.^23^ In contrast, conventional NiTi wires usually present Rs values near 10°C, which keeps them fully austenitic during clinical use (Figure 2) and results in lower flexibility but higher superelastic behavior.^23^

### Mechanical and clinical consequences of heat treatment

Heat-treated NiTi instruments generally show greater flexibility and higher resistance to cyclic fatigue than conventional alloys. When the alloy contains a higher martensitic fraction, as in Controlled-Memory, Gold, and Blue wires, the instruments tolerate larger deflections and resist fatigue better than fully austenitic instruments.^4,17^ This metallurgical effect is evident in comparative studies, in which Reciproc Blue (VDW), despite sharing the same geometry as Reciproc (VDW), exhibited higher flexibility and greater fracture resistance, confirming the strong influence of the alloy phase on mechanical behavior.^24,26^ These mechanical advantages have clinical consequences.

Martensitic instruments facilitate safer negotiation of curved canals by combining enhanced flexibility with improved canal centering, thereby reducing the risk of canal transportation.^27^ These properties may also contribute to greater cutting efficiency,^28^ a lower incidence of instrument separation,^20^ and extended instrument lifespan.^26^ In contrast, austenitic instruments generally exhibit greater resistance to torsional stress because they require higher torque to reach fracture, which is particularly relevant under taper-lock conditions and other high-torque scenarios.^24^ In addition, clinical performance does not depend on metallurgy alone but results from the interaction between alloy phase and instrument design. Features such as cross-sectional design, tip size, taper, and core diameter directly influence stress distribution and cutting dynamics. For example, a larger core diameter increases torsional resistance while simultaneously reducing flexibility, thereby creating a trade-off between mechanical strength and canal-centering ability.^29^ More recently, multi-heat-treatment systems such as ProTaper Ultimate (Dentsply Sirona) and Genius ProFlex (Medidenta)^30^ have integrated modified metallurgy with refined geometric design to balance flexibility, cutting efficiency, and mechanical strength in accordance with specific shaping objectives.^31^

For practitioners, knowledge of metallurgy has direct clinical relevance. Understanding phase transformation temperatures and interpreting laboratory tests, such as DSC, help predict instrument behavior and reinforce the role of heat treatment in modern NiTi instrument design.^17,22,26^

### Instrument geometry and design innovations

**Figure 3:**
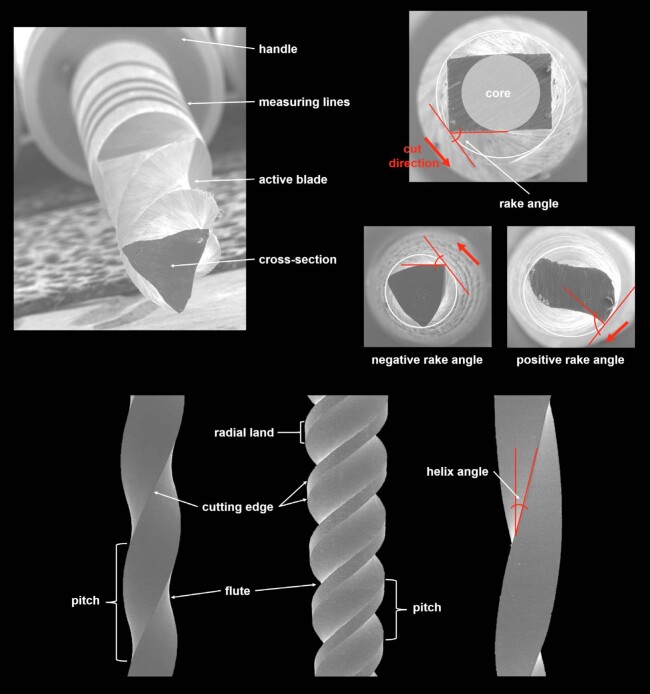
Schematic representation of the main design features of endodontic mechanical instruments. The illustration highlights the cutting edges and intervening flutes responsible for dentin removal and debris collection, as well as the pitch and helix angle, which influence flexibility, stress distribution, and debris transportation. Radial lands (marginal widths) connecting the cutting edges at the periphery are shown, emphasizing their role in limiting canal transportation while increasing contact area and friction. The central core diameter, related to flute depth, is illustrated as a key determinant of resistance to cyclic fatigue and torsional stress. Cross-sectional views demonstrate variations in rake angle and the presence of multiple cutting edges, which modulate cutting aggressiveness and contact mechanics.

Endodontic mechanical instruments do not follow a single regulatory standard, but their main design features are readily identifiable. The cutting edges form the active part of the instrument and contact the root canal walls to remove superficial dentin during shaping. Between these edges lies the flute, which is present only in the active region and collects dentin chips and pulp remnants during rotation or reciprocation. Flute performance depends on its depth, width, geometry, and surface finish, which govern debris space and flow. Pitch refers to the distance between matching points on adjacent spirals. A smaller pitch increases the number of spirals along the blade and improves flexibility. Pitch can remain constant or vary along the active length to modify stress distribution and debris transport. Radial lands, also referred to as marginal widths, form flattened surfaces that connect the cutting edges at the periphery and help limit canal transportation and reduce the screw-in effect. However, by increasing the contact area between the instrument and dentin, radial lands also increase frictional resistance, which may reduce cutting efficiency and potentially increase torsional stresses under clinical conditions. The helix angle, defined as the angle between the long axis of the instrument and the cutting edge, affects the direction and speed of debris removal. At the center of the instrument, the cylindrical core has a diameter that corresponds to flute depth and strongly influences resistance to cyclic fatigue and torsional stress. Some instruments use a core taper smaller than the taper of the cutting flutes, which allows deeper flute formation in the coronal region and improves debris storage. The rake angle, visible in cross-section, describes the angle between the cutting edge and the instrument radius and can be positive or negative, which modifies cutting aggressiveness. Some cross-sections show more than one cutting edge, and each edge may display a different rake angle, which alters contact mechanics and cutting behavior^14^ (Figure 3).

Changes in design features strongly influence instrument performance and clinical behavior by altering stress distribution along the instrument during shaping. Torsional resistance in straight canals increases with cross-sectional area, whereas resistance to cyclic fatigue in curved canals decreases as cross-sectional area increases, creating a mechanical trade-off. As the contact area between the instrument and dentin increases, the torque required for rotation also rises. Consequently, instruments with a smaller pitch and a higher number of spirals, or with radial lands, require higher torque during use. In contrast, instruments with fewer contact points or sharper cutting edges reduce contact area, achieve effective cutting at lower torque, and often require a higher rotational speed to maintain cutting efficiency. Additionally, a lower number of spirals reduces the tendency to cause a screw-in effect. Instruments with greater flexibility and asymmetric cross-sectional designs further reduce canal transportation during shaping by improving canal-centering ability. This increased flexibility usually results from an increased number of spirals, although this change reduces torsional strength. Conversely, more rigid instruments tend to exhibit fewer spirals and a larger core diameter, which increases torsional resistance but limits flexibility, reinforcing the balance between strength and shaping safety.^14^

Beyond conventional design parameters, manufacturers have introduced flat-side and hybrid blade geometries. Systems such as AF F One Blue (Fanta Dental Material Co.), Platinum V.EU (Bondent), and Flash Endo Power (Bondent) incorporate these designs. These configurations aim to reduce the contact area, increase the space for debris, and improve cutting efficiency. However, current evidence does not confirm consistent clinical advantages, and the impact on shaping quality, safety, and debris removal remains uncertain.^32-36^

**Figure 4:**
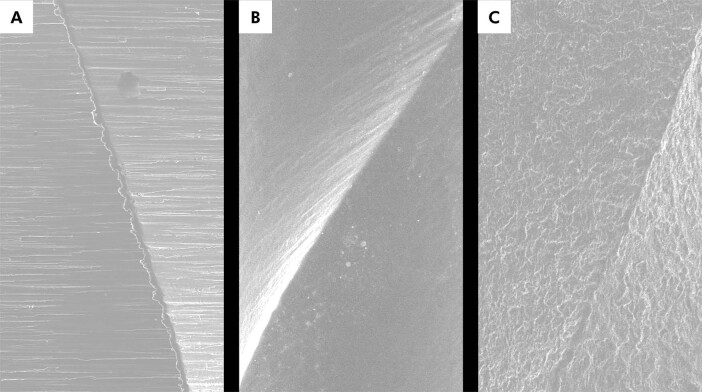
Representative scanning electron microscopy images of three distinct surface finishes. (a) A typical surface of a WaveOne Gold (Dentsply Sirona) instrument shows the characteristic parallel marks produced by the manufacturing grinding process; (b) A One Curve Mini (MicroMega) instrument displays a smoother surface with fewer irregularities, resulting from electropolishing; (c) On the right, an electric discharge machining (EDM) surface, as employed in the HyFlex EDM (Coltene-Whaledent) manufacturing process, reveals a markedly irregular topography.

Microscopic surface features also affect instrument performance because they influence crack initiation and propagation at the metal surface. Traditional manufacturing often leaves parallel grinding marks (Figure 4), which act as stress concentrators and promote early crack formation under cyclic loading. Electropolishing smooths surface irregularities by removing superficial defects (Figure 4), which improves flexibility^37^ and delays crack initiation, thereby increasing resistance to cyclic fatigue^38^. Electrical discharge machining represents another surface modification strategy, as used in HyFlex EDM (Coltene-Whaledent). This noncontact process uses controlled electrical discharges in a dielectric fluid to erode the metal and create a highly irregular surface topography (Figure 4).^39,40^ This surface morphology reduces internal residual stresses and enhances phase distribution, which may explain the greater flexibility and higher cyclic fatigue resistance reported for EDM-treated NiTi instruments. A further surface treatment involves a DLC coating, as applied in the RCS Rainbow system (Ramo Medical). This process deposits a thin, hard carbon film on the instrument surface, increasing surface microhardness, reducing friction against dentin, and improving flexibility and cyclic fatigue resistance compared with uncoated instruments.^25^

### Shaping outcomes and clinical performance

In endodontics, the shaping ability of mechanical NiTi instruments refers to their capacity to produce a continuous, centered, and biologically appropriate root canal geometry that follows the original canal anatomy while achieving sufficient enlargement for effective irrigation and obturation, with minimal procedural errors and structural compromise.

Shaping ability includes maintaining canal trajectory by respecting natural curvatures and minimizing canal transportation, ledging, zipping, and straightening, thereby producing a preparation that remains centered along its length. It also involves controlled and selective dentin removal that creates a smooth tapered preparation while preserving pericervical and radicular dentin, particularly in thin or highly curved roots. In addition, shaping ability includes generating a continuous and reproducible taper with gradual, consistent conicity from the apical to the coronal region, compatible with effective irrigant penetration and sealer distribution, and free of abrupt procedural irregularities. Shaping performance is strongly influenced by instrument metallurgy, cross-sectional design, taper, tip configuration, flexibility, and resistance to torsional and cyclic fatigue, which collectively determine how predictably the instrument adapts to canal anatomy and how safely it enlarges the canal without deformation or separation. Shaping ability is most commonly assessed by comparing postoperative canal geometry with preoperative anatomy, typically using micro-computed tomography (micro-CT) analysis of canal centering ratio, canal transportation, volumetric changes, percentage of unprepared canal walls, and remaining dentin thickness. This assessment represents a multidimensional concept that reflects how effectively and safely a complex and irregular root canal can be transformed into a well-centered, smoothly tapered, and biologically functional shape while preserving the structural integrity and spatial configuration of the root.

### Canal transportation

Preservation of the original root canal anatomy remains a fundamental objective of contemporary endodontic therapy because canal transportation has been consistently associated with procedural errors and compromised treatment outcomes.

**Figure 5:**
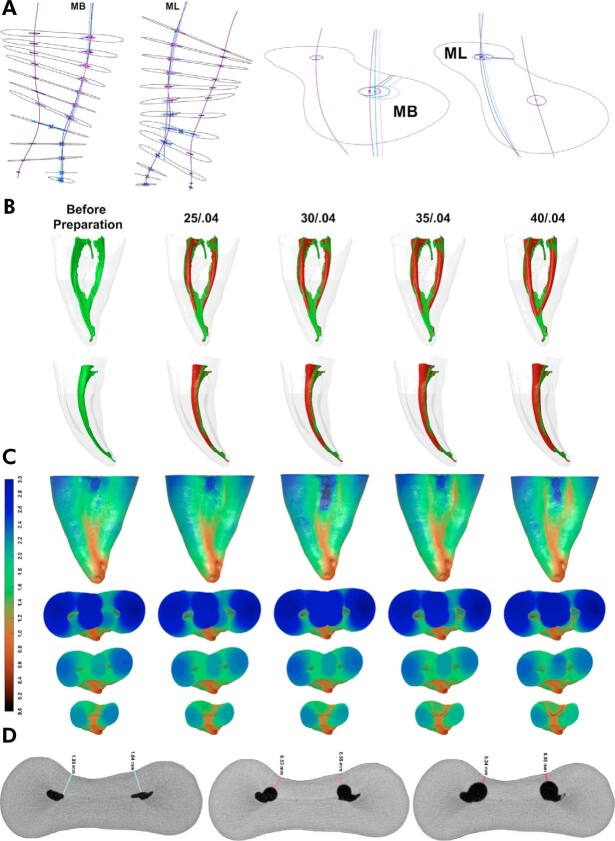
(a) Automated computational workflow for digital image analysis based on micro-CT datasets used to quantify canal transportation in each axial slice by measuring the linear displacement of the geometric center of the mesiobuccal (MB) and mesiolingual (ML) canals of a mandibular molar before preparation (purple) and after rotary instrumentation to sizes 30/.04 (blue) and 30/.06 (pink); (b) 3D reconstructions of two mesial roots of mandibular molars before and after four sequential preparation steps using a rotary system. The red overlay indicates the progressive increase in canal volume with enlargement, whereas green indicates the unprepared root canal space; (c) Color-coded thickness maps corresponding to the roots shown in (b), illustrating changes in root dentin thickness after instrumentation; thicker areas are represented in blue and green, while thinner dentine regions are shown in red; (d) Representative axial cross-sections of a mesial root of a mandibular molar demonstrating the progressive reduction in dentin thickness on its distal aspect following sequential enlargement with reciprocating instruments.

Transportation, defined as the deviation of the prepared canal from its original path, may result in excessive dentin removal in critical areas, including furcal and external danger zones, thereby increasing the risk of strip perforation and impairing the ability to achieve an effective apical seal (Figure 5a).^41^ The advent of NiTi instrumentation represented a major advance over stainless-steel files by substantially reducing the incidence and severity of transportation, particularly in curved canals, because of the superior flexibility and superelasticity of NiTi alloys.^41,42^

Within the currently available NiTi systems, a central debate concerns the influence of kinematics—specifically continuous rotary versus reciprocating motion—on shaping performance and canal transportation. Evidence derived from micro-CT studies and systematic reviews suggests that continuous rotary systems tend to demonstrate superior centering ability and reduced canal transportation compared with reciprocating systems, particularly in moderately and severely curved canals.^41,43-46^ This advantage is most consistently observed in the middle and coronal thirds of the canal, whereas the apical third often exhibits comparable performance across different systems and motions.^43,47,48^ Continuous rotary instruments such as ProTaper Next (Dentsply Sirona) have repeatedly shown lower transportation values and more centered preparations than reciprocating systems such as Reciproc (VDW) and WaveOne Gold (Dentsply Sirona),^43,46^ likely because uninterrupted rotational movement produces more uniform dentin removal. However, studies have reported mixed findings. A systematic review highlighted that when a broad range of instruments and alloys is considered, reciprocating single-file and continuous rotary systems may demonstrate similar transportation and centering ability.^49^ This inconsistency indicates that kinematics alone does not fully explain shaping outcomes. Instead, instrument design parameters—including taper, cross-sectional geometry, and thermomechanical treatment of the NiTi alloy—appear to exert a substantial influence on transportation.^42,49-52^ Instruments manufactured from controlled-memory or other heat-treated alloys exhibit enhanced flexibility, which may partly explain the improved shaping performance observed in certain rotary systems.^41^

Moreover, not all reciprocating systems perform equivalently. Recent evidence suggests that specific reciprocating designs, such as R-Motion, may better preserve canal anatomy and limit unnecessary dentin removal compared with other reciprocating instruments.^53^ Similarly, adaptive motion systems such as Twisted File Adaptive (SybronEndo) have been reported to reduce apical transportation compared with traditional reciprocating kinematics,^50-52^ suggesting that hybrid or adaptive motions may offer advantages in critical apical regions. Nevertheless, no currently available system completely eliminates canal transportation, and untouched canal walls remain a consistent finding across different instruments and kinematics.^41,42,51^

Procedural strategies also play a relevant role in transportation control. Coronal preflaring has been shown to reduce torsional stress on instruments and improve centering ability in certain rotary systems, thereby indirectly limiting canal deviation.^54^ In addition, establishing a reproducible glide path before NiTi instrumentation is considered an essential step and significantly decreases the risk of transportation and instrument fracture, regardless of the kinematic approach used.^43^ These findings reinforce the concept that shaping outcomes are multifactorial and depend on the interaction between instrument design, alloy properties, motion, and operator-controlled technique.

In summary, the available evidence indicates that continuous rotary NiTi systems are generally associated with less canal transportation and superior centering ability than reciprocating systems, particularly in curved canals and in the middle and coronal thirds of the root canal.^41-46^ Differences in the apical third are less pronounced, with many systems demonstrating comparable performance in this critical area.^43,47,48^ Instrument design, alloy heat treatment, and adherence to sound clinical protocols, including glide path creation and coronal flaring, appear to be as influential as kinematics in minimizing transportation. The main finding supported by current data is that modern continuous rotary NiTi systems tend to provide more predictable shaping with reduced transportation. However, no system completely prevents canal deviation, and clinical success remains dependent on appropriate case selection and proper technique.

### Volumetric changes

Quantitative assessment of volumetric changes has become central to understanding the shaping efficacy of NiTi instruments, particularly with the increasing use of micro-CT as the reference standard for three-dimensional analyses of canal geometry. The literature consistently supports the view that effective cleaning and disinfection are associated with sufficient increases in canal volume and surface area, which enhance irrigant penetration and exchange while creating space for obturation. Furthermore, excessive dentin removal is undesirable because of its negative impact on root strength and long-term structural integrity.^49,55^

The available evidence regarding the influence of kinematic motion on volumetric outcomes remains inconclusive. Some studies and systematic reviews indicate that reciprocating systems may be associated with greater volumetric enlargement and surface area increase than continuous rotary systems, potentially because of their intermittent cutting action and distinct engagement with canal walls.^55^ In contrast, another review reported no statistically significant differences between rotary and reciprocating kinematics when volumetric outcomes are considered, suggesting that final apical size, taper, cross-sectional design, and alloy thermomechanical treatment exert a stronger influence than motion alone.^49^

Recent conceptual developments have further refined the interpretation of volumetric changes by shifting the focus from simple geometric enlargement to the concept of functional endodontic volume. This framework emphasizes that the clinically relevant outcome of shaping is not merely the amount of dentin removed but the creation of a biologically effective three-dimensional space that optimizes irrigant flow, replacement, and biofilm disruption while preserving tooth structure.^56^ Within this perspective, volumetric changes induced by different NiTi systems should be analyzed in terms of their functional impact on irrigation dynamics rather than absolute numerical values. Nevertheless, this concept requires further experimental and clinical validation.

Micro-CT studies have shown that even large apical enlargement leaves a substantial portion of unprepared canal walls, indicating that increasing canal volume alone does not achieve complete mechanical cleaning.^57^ The design and metallurgical properties of NiTi instruments influence the geometry and surface topology of prepared canals,^23,28,30,35,39,58,59^ which in turn modulate fluid dynamics within the canal system. These findings support the notion that volumetric outcomes must be interpreted alongside qualitative changes in canal morphology. Another relevant aspect is the interaction between the moving instrument, the irrigant, and the canal wall. Hydrodynamic shear stresses generated during instrumentation can enhance biofilm disruption, and these effects depend not only on the achieved volume but also on canal geometry, fluid properties, and kinematics.^60,61^

From a clinical perspective, these data imply that volumetric enlargement produced by NiTi instruments should be viewed as a means to an end, namely improved disinfection, rather than as an isolated technical objective. Conservative shaping strategies that selectively increase volume in critical regions, such as the apical third and isthmuses, may offer a more favorable balance between cleaning efficacy and dentin preservation, consistent with minimally invasive principles. The integration of advanced irrigation technologies further reduces the need for excessive volumetric enlargement, provided that an adequate functional space for irrigant dynamics is created.

Based on available data, NiTi instruments consistently promote significant volumetric enlargement of root canals regardless of kinematic motion. Although some evidence suggests that reciprocating systems may produce slightly greater volumetric and surface area increases, the preponderance of evidence indicates that these differences are not clinically meaningful. The principal determinant of effective shaping appears to be the creation of a functional three-dimensional canal volume that optimizes irrigant dynamics while preserving dentinal structure, rather than the specific motion of the instrument.

### Unprepared canal walls

Unprepared canal walls represent one of the most persistent limitations of contemporary endodontic instrumentation. Despite substantial advances in rotary and reciprocating NiTi systems, mechanical preparation alone remains unable to access and shape the entire root canal surface, particularly in anatomically complex regions such as oval extensions, fins, isthmuses, and apical ramifications.^62^ Micro-CT analyses have consistently demonstrated that a considerable proportion of the canal surface area remains untouched following instrumentation, independent of the alloy, thermal treatment, instrument design, or kinematic strategy (Figure 5b).^23,27,30,35,63-65^

Quantitative evidence indicates that untouched canal wall areas in molar teeth commonly range from 18% to 35%, with higher values reported in curved canals and in the apical third.^45,48,59^ These findings highlight the anatomical constraints that inherently limit the ability of endodontic instruments to conform to irregular canal geometries. Although increasing final apical preparation size and taper reduces the proportion of untouched surfaces, it does not achieve complete elimination, even with larger apical preparations.^66^ This observation reinforces the concept that canal anatomy, rather than instrument kinematics alone, exerts a dominant influence on the extent of surface contact during preparation.^63^

The comparison between continuous rotary and reciprocating motions has generated considerable debate. Some individual studies have suggested that continuous rotation may produce more uniform wall contact, particularly in the middle third of the canal, possibly because of a more consistent cutting trajectory and sustained dentin engagement.^55^ However, systematic reviews and meta-analytical evidence indicate that the differences between these kinematics are neither robust nor consistently reproducible across different systems and experimental models.^49^ Most studies report comparable percentages of untouched canal walls between rotary and reciprocating instruments, suggesting that the motion itself exerts a secondary effect compared with canal morphology and preparation dimensions.^45,48,53,59,67^

The clinical implications of unprepared canal walls are supported by histological and scanning electron microscopy observations, which demonstrate that these areas frequently harbor residual pulp tissue, smear layer, dentinal debris, and viable bacteria, particularly in the apical third.^68^ These findings provide a biological rationale for persistent apical periodontitis and post-treatment disease, even in cases in which shaping appears radiographically satisfactory. Therefore, mechanical preparation should be viewed primarily as a facilitator of effective chemical debridement rather than as an independent cleaning modality.

From a microbiological perspective, studies have consistently shown that areas not contacted by instruments may retain structured bacterial biofilms that are less susceptible to irrigants, particularly in complex apical anatomy. Although both hand and engine-driven instrumentation significantly reduce intracanal bacterial load, neither approach consistently achieves complete microbial elimination when used alone, even when assessed by sensitive molecular methods. Clinical comparisons between manual stainless-steel instrumentation and engine-driven NiTi systems have generally reported comparable overall levels of bacterial reduction, although some culture-based and PCR analyses suggest fewer positive cases after mechanized preparation.^69^ This apparent similarity may be partly explained by methodological limitations inherent to intracanal microbiological sampling, which tends to recover microorganisms primarily from instrumented or irrigant-accessible areas, potentially underrepresenting biofilms located in untouched recesses of the root canal system. Nevertheless, viable microorganisms are frequently detected after both manual and mechanized shaping, reinforcing the concept that mechanical instrumentation primarily reduces microbial burden and facilitates chemical disinfection rather than achieving sterility.

In this context, adjunctive irrigation strategies assume critical importance. The creation of sufficient canal space through shaping is essential to allow irrigant penetration, fluid exchange, and activation within anatomical complexities that instruments cannot physically contact.^62^ Activated irrigation techniques, improved irrigant delivery systems, and contemporary protocols aimed at enhancing hydrodynamic effects are therefore indispensable components of modern endodontic therapy.

In conclusion, available evidence demonstrates that a substantial proportion of canal walls remain untouched after preparation with both rotary and reciprocating NiTi instruments. These untouched areas are primarily determined by the original canal anatomy and final preparation dimensions rather than by the specific kinematic motion. Therefore, neither rotary nor reciprocating instrumentation can achieve complete mechanical debridement, underscoring the indispensable role of optimized chemical cleaning and irrigant activation for effective disinfection of the root canal system.

### Remaining dentin thickness

Preservation of remaining dentin thickness after root canal preparation has emerged as a central biological and mechanical principle in contemporary endodontics, given its direct relationship with the long-term structural integrity and fracture resistance of treated teeth (Figure 5c–d). Excessive dentin removal, particularly in the pericervical region and so-called danger zones, has been consistently associated with increased susceptibility to vertical root fracture and catastrophic structural failure.^70-73^ In this context, the development of NiTi instruments has represented a major advancement over stainless steel files by enabling more centered preparations and better preservation of native canal anatomy, thereby reducing unnecessary dentin removal.

Current evidence demonstrates that remaining dentin thickness is primarily determined by instrument design characteristics rather than kinematics alone. Parameters such as taper, cross-sectional geometry, metallurgical treatment, and cutting efficiency exert a stronger influence on dentin removal than whether the system operates in continuous rotation or reciprocation.^7,17,72^ Micro-CT studies, regarded as the gold standard for three-dimensional assessment of dentin thickness and canal geometry, consistently show that reciprocating systems with larger tapers, including WaveOne Gold (Dentsply Sirona) and Reciproc Blue (VDW), tend to remove more dentin than rotary systems with smaller and more conservative tapers, particularly in the pericervical region and in high-risk areas of curved roots.^53,67,74^ In contrast, minimally tapered rotary systems such as TruNatomy (Dentsply Sirona) and XP-endo Shaper (FKG) demonstrate superior dentin preservation while maintaining canal centricity and anatomical integrity.^53,67,74-77^

Despite these differences in dentin removal patterns, most contemporary NiTi systems appear capable of maintaining remaining dentin thickness within clinically acceptable thresholds. Multiple micro-CT investigations indicate that the minimal remaining dentin thickness after preparation generally remains above 0.5 mm in critical regions, which is considered a safety margin that reduces the risk of strip perforation and structural weakening, independent of the system used.^74,76^ Nevertheless, the magnitude and distribution of dentin removal are not uniform. Reciprocating instruments with greater tapers, particularly those reaching 0.08, have been associated with a higher percentage of dentin reduction on the distal aspect of mesial roots of mandibular molars, an area classically recognized as highly vulnerable to thinning and iatrogenic damage.^75^

The role of kinematics in preserving remaining dentin thickness appears secondary to system-specific design. Although traditional assumptions suggested that reciprocation might be inherently more aggressive, recent *ex vivo* studies indicate that this is not universally true. Certain reciprocating systems, such as One Reci (MicroMega), have been shown to produce less pericervical dentin wear than some rotary systems, including WaveOne Gold (Dentsply Sirona), highlighting that preparation protocol and taper strategy are more determinative than motion itself^78^. These findings reinforce the concept that conservative coronal shaping and the use of minimal-taper designs are important for dentin preservation, irrespective of whether the instrument is driven in continuous rotation or reciprocation.

Another critical aspect influencing remaining dentin thickness is the relationship between dentin removal and canal transportation. Instruments with superior centering ability and reduced transportation tend to preserve more uniform dentin thickness by avoiding excessive cutting on the outer walls of curved canals and in danger zones.^49^ This may explain why systems that maintain the original canal trajectory and exhibit flexible, adaptive designs are consistently associated with higher remaining dentin thickness values. Furthermore, conservative access cavity designs, although conceptually appealing, have not been shown to significantly alter remaining dentin thickness compared with traditional access cavities. In contrast, root-specific anatomy, such as differences between mesial and distal roots, plays a more decisive role in local dentin thickness variations.^75^

From a clinical perspective, these findings suggest that the preservation of remaining dentin thickness should be addressed through a multifactorial strategy that includes conservative access, selection of low-taper instruments, and careful control of the final apical preparation size. Evidence indicates that, when used appropriately, most modern NiTi rotary and reciprocating systems achieve adequate apical preparation quality without compromising critical dentin thickness.^76^ However, rotary systems with minimal taper continue to be favored in the context of minimally invasive endodontics because of their consistent performance in maximizing dentin preservation while maintaining shaping efficacy.^53,67,74-76^

Based on the available evidence, remaining dentin thickness after root canal preparation is predominantly influenced by instrument taper and design rather than by kinematics alone. Although both rotary and reciprocating NiTi systems generally maintain remaining dentin thickness within clinically acceptable limits, systems with more conservative tapers demonstrate superior dentin preservation, particularly in the pericervical and danger-zone areas.

### Clinical performance

NiTi instruments have consistently demonstrated superior clinical performance compared with traditional stainless-steel hand files, particularly in terms of flexibility, resistance to cyclic and torsional fatigue, and shaping predictability. Contemporary systems manufactured with advanced heat treatments and modified alloy compositions exhibit enhanced mechanical behavior, reducing the incidence of instrument fracture and limiting canal transportation during root canal preparation.^1,7,20,79-81^ Heat-treated martensitic NiTi alloys, including Gold, Blue, and Controlled-Memory wire technologies, show increased flexibility and fatigue resistance relative to conventional austenitic alloys, translating into safer and more controlled preparation of curved and anatomically complex canals.^1,7,80^

Evidence from systematic reviews and meta-analyses suggests that engine-driven NiTi preparation improves the technical quality of root canal treatments by increasing the frequency of adequately shaped and filled canals and reducing the occurrence of ledges, without a concomitant increase in major procedural errors, such as perforations or instrument separation.^1,82^ These advantages are particularly evident in undergraduate and less experienced clinical settings, in which rotary and reciprocating NiTi systems consistently outperform stainless-steel hand files in terms of procedural quality and reproducibility.^82^ Nonetheless, the overall certainty of this evidence remains limited because of methodological limitations and heterogeneity among available studies.

When long-term clinical outcomes are considered, the relationship between NiTi technology and periapical healing is more complex. A recent systematic review and meta-analysis reported that contemporary instrumentation techniques, including engine-driven NiTi systems, were associated with significantly higher odds of radiographic healing compared with stainless-steel instrumentation, with an odds ratio of 2.07 and a minimum follow-up of 1 year.^2^ However, the included studies were characterized by low methodological quality and high risk of bias, and no evidence was found to support significant differences in healing rates among different NiTi systems. Retrospective clinical data also suggest higher healing rates in molars prepared with NiTi rotary instruments compared with stainless-steel hand files, with reported healing rates of 77% versus 60% and fewer procedural errors in the NiTi groups.^83^ These findings indicate a general clinical benefit of NiTi technology over traditional hand instrumentation rather than superiority of any specific system.

Observational cohort studies provide further insight into the determinants of long-term healing. Large-scale clinical cohorts evaluating various NiTi rotary systems have reported favorable healing rates approaching 87% at medium-term follow-up; however, multivariate analyses consistently demonstrate that outcomes are primarily influenced by biological and case-related factors, including the presence of preoperative periapical lesions and retreatment status, rather than by the specific NiTi system or obturation technique employed.^2,83,84^ Systematic reviews of cohort data similarly conclude that, although contemporary NiTi instrumentation is associated with improved healing compared with older techniques, compelling clinical evidence supporting meaningful differences is lacking in long-term outcomes among various NiTi designs.^84^

In summary, contemporary NiTi instrument systems represent the current standard of care in root canal preparation because of their superior mechanical properties, improved shaping outcomes, and association with higher radiographic healing rates compared with stainless-steel hand instrumentation. However, current clinical evidence does not support clinically significant differences in long-term healing of apical periodontitis among different NiTi systems. The primary clinical significance of NiTi technology lies in its ability to provide safer, more predictable, and anatomically respectful canal preparation, thereby enhancing the technical quality of treatment. Long-term success remains predominantly determined by preoperative periapical status, case complexity, and overall biological and procedural factors, underscoring the need for further high-quality prospective clinical studies to clarify the relative contribution of specific instrument designs to treatment outcomes.

### Mechanical properties and failure mechanisms

The mechanical behavior of NiTi endodontic instruments depends on the combined effect of alloy state, macro-geometry, surface condition, and kinematics.^3,4,23,28,39,58,59^ Alloy state determines phase composition at the temperature of use, thereby influencing the elastic and plastic response of the material. Macro-geometry, defined by tip size, taper, core mass, cross-sectional design, and helical parameters, controls stress distribution along the instrument during bending and rotation. Surface condition, which depends on machining quality, residual stresses, oxide layer, and post-processing, such as electropolishing, electrical discharge machining, and surface coatings, influences friction, crack initiation, and wear. Kinematics, through the type of motion and rotational speed applied during use, further modifies stress accumulation and fatigue patterns. Compared with stainless steel, NiTi exhibits a lower effective elastic modulus and reduced springback, particularly when martensite or R-phase prevails at intracanal temperatures, which allows larger recoverable strain and improves tracking of canal curvature.

In curved canals, these properties reduce centering errors for a given tip size and taper but do not eliminate the basic trade-offs that govern bending, cutting, and failure. Additionally, surface condition influences friction against dentin, which increases torque demand and affects notch sensitivity and crack initiation under cyclic loading. During bending, flexibility depends mainly on alloy stiffness at the temperature of use and on the amount of metal in the section that follows the canal curvature. For a given curvature, a thicker core or a higher local taper experiences greater tensile strain at the outer surface, increasing elastic recovery and pushing the instrument away from the canal center. In contrast, a reduced core diameter lowers surface strain, improves centering, and allows improved tactile control and canal guidance. Designs that reduce continuous wall contact can moderate instrument engagement; however, bending and centering in clinical use depend primarily on the combined effect of alloy phase state and local core mass. When heat treatment produces a predominantly martensitic structure but the core remains bulky, geometry usually sets the practical limit for flexibility and centering ability.

Buckling represents axial column instability during apical advancement. When compressive load on a slender, highly flexible instrument exceeds its column strength, the instrument deflects laterally and loses cutting ability.^85^ This event occurs more frequently with small-core designs used in long, narrow, or poorly negotiated canals. It may also result from inadequate straight-line access and excessive apical pressure. Because buckling results from an imbalance between applied load and structural resistance, prevention and management depend on controlling mechanical demands during instrumentation. Prevention relies on controlled mechanical loading throughout shaping. Clinicians should establish a reproducible glide path and remove coronal interferences when indicated, then advance the instrument with light apical pressure and short strokes of approximately 1 to 3 mm. Periodic withdrawal allows cleaning of flutes and renewal of the irrigant, which limits debris compaction and friction. When buckling appears, the operator should reduce compressive demand by confirming canal patency, refining the glide path, and shortening the stroke amplitude before further advancement. If instability persists, the use of an instrument with greater column strength is recommended for the corresponding stage of canal preparation.^4^

Cutting ability describes how an instrument contacts dentin and removes debris. Rake angle, land design, flute depth, and helix angle govern the balance between dentin removal and friction. As contact area increases because of a larger core, greater taper, or prolonged engagement at the same depth, torque demand and heat generation increase.^4^ When debris accumulates inside the flutes, cutting efficiency decreases and the risk of taper-lock increases. Therefore, cutting performance depends strongly on technique. Short, controlled pecking motions with frequent withdrawal and cleaning of the flutes, combined with active irrigation and lubrication, preserve cutting efficiency and reduce mechanical load associated with instrument failure.^18,28,35,39^ These mechanical interactions also explain why cutting efficiency differs between forward progression and sidewall contact.

Axial cutting advances the instrument apically, while lateral cutting removes dentin along the canal wall. Axial cutting occurs when cutting edges engage dentin and chip space allows debris to escape, such that light apical pressure results in the removal of dentin. This mechanism fails when the instrument is too slender or flexible to resist compressive forces, leading to buckling and surface rubbing rather than cutting. Lateral cutting becomes dominant in curved canal segments and during brushing movements. It increases with elastic recovery and greater local core mass or taper, both of which increase wall contact, torque demand, and heat production^4^. In contrast, an instrument with a smaller core and greater flexibility reduces lateral contact and improves centering because of improved bending behavior, but it may lose axial efficiency in narrow paths if compressive support is insufficient. Safe and efficient cutting results from balancing these effects. Clinicians should use short axial pecking motions with frequent withdrawal to clear flutes and renew irrigant. Lateral brushing should serve to remove coronal interferences rather than to force apical progression in curved regions. Instrument geometry and alloy should provide enough axial stiffness to prevent buckling while limiting unnecessary lateral load. Prolonged instrument contact at a fixed depth increases engagement and allows debris compaction inside the flutes, promoting taper-lock. Active irrigation and lubrication reduce friction and heat, helping maintain cutting efficiency and lowering mechanical stresses that can lead to failure. NiTi endodontic instruments primarily fracture due to torsional overload or cyclic fatigue.^86^ Although these failure modes often occur independently, they can also interact during clinical use.

**Figure 6:**
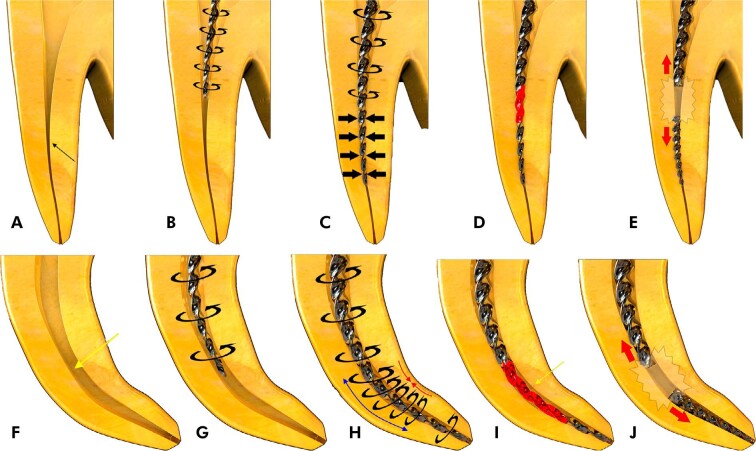
Schematic representation of rotary NiTi instrument fracture during clinical use. (a-e) Torsional failure: (a-b) The instrument rotates continuously at constant speed and torque in a narrow canal (arrow). (c-d) When the tip (or another segment) binds at the narrowest point, the shank continues rotating. (e) Exceeding the elastic limit due to applied torque leads to inevitable fracture. (f-j) Cyclic fatigue: (f-g) The instrument rotates continuously at constant speed and torque in a curved canal (arrow). (h-i) At the curvature, the outer side of the shaft is subjected to tension and the inner side to compression. (j) Repeated tension-compression cycles at the point of maximum flexure promote microcrack formation and propagation, resulting in catastrophic fracture due to metal fatigue.

**Figure 7:**
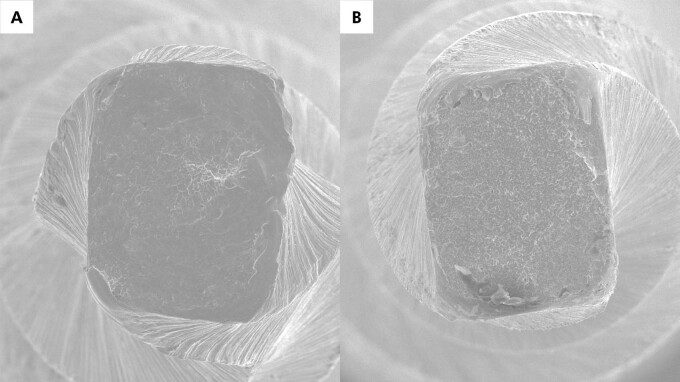
Representative scanning electron microscope images of NiTi instrument cross-sections with torsional failure (a) and cyclic fatigue failure (b). Torsional failure typically presents as a smooth peripheral area surrounding a central zone characterized by dimpling features on the fracture surface, whereas cyclic fatigue failure is usually marked by a widespread distribution of dimpling across the entire fracture surface.

Torsional failure occurs when the tip of the instrument binds inside the canal while the shank continues to rotate (Figure 6a–e). This condition generates intense torsional stress over a short period. When shear strain exceeds the elastic limit of NiTi, which is approximately 8%, the alloy undergoes plastic deformation and then fractures.^15^ Instruments that fail by torsion commonly show unwinding of the flutes, indicating prior plastic deformation^86^. Scanning electron microscopy shows a smooth peripheral zone surrounding a central area with dimples and microvoids, which are typical of ductile fracture^87^ (Figure 7). These features represent the final stage of material separation after continued rotation at the binding point. The main clinical cause of torsional failure is instrument locking in a narrow or curved canal while the motor continues to apply torque.^88^ This risk increases when wall contact rises, dentin debris packs inside flutes, or the operator applies excessive apical force. These conditions increase torsional stress and accelerate fracture.^89,90^

Cyclic fatigue occurs when a NiTi instrument undergoes repeated tensile and compressive stresses during rotation in curved canals or during prolonged use under low external load (Figure 6f–j). These cyclic stresses initiate microcracks that progressively propagate through the alloy until fracture occurs.^91,92^ Scanning electron microscopy of fatigue fractures shows widespread dimpling across the fracture surface, with microvoids and characteristic crack patterns^87^ (Figure 7). In contrast with torsional failure, fatigue fractures do not show spiral unwinding or other signs of plastic deformation. As cyclic loading continues, the instrument loses its capacity to tolerate the original elastic strain limit, increasing the risk of sudden separation. Under clinical conditions, fracture often results from the combined action of cyclic fatigue and torsional stress, particularly when a rotating instrument alternates between binding and release in curved canal segments.^88^

## Safety considerations

Safe mechanical root canal preparation relies on managing instrument load throughout the procedure by minimizing unnecessary contact and torque, limiting time in curved regions, and selecting instruments with alloy properties and geometry suited to canal anatomy. Following these guidelines, together with proper technique, reduces the risk of the two main failure modes: torsional overload and cyclic fatigue.^93,94^ Effective load management begins with careful preoperative planning and case selection. Curvature severity, canal length, calcifications, and canal diameter should be assessed using preoperative imaging, and straight-line access should be planned to fully expose the canal orifice and the coronal third. The smallest tip size and taper that accomplish the shaping goal should be selected to minimize contact, torque, and the risk of instrument failure. A NiTi system and motion should be selected based on canal anatomy: heat-treated, more flexible instruments with reciprocation or adaptive motion may be preferable in severe curvatures or narrow canals, whereas continuous rotation is generally safe in less constricted canals previously enlarged with glide path instruments.

Glide path creation and coronal management are essential for safe instrumentation because they establish the foundation for controlled mechanical work. Clinicians should begin by creating a reproducible glide path that freely accepts a hand file of appropriate size before introducing rotary NiTi instruments. Coronal interferences should be relieved carefully to prevent taper-lock deeper in the canal while preserving dentin. Patency should be maintained, and recapitulation performed regularly, using active irrigation to suspend debris and reduce friction, thereby facilitating smooth instrument progression along the canal.

Mechanical load also depends on motor control and preparation protocol. Clinicians should use a torque-controlled motor at manufacturer-recommended settings, treating these values as maximum limits rather than targets. Instruments should be advanced with light apical pressure in short strokes of approximately 1 to 3 mm, with frequent withdrawal to clean flutes and refresh the irrigant. Prolonged rotation at a fixed depth should be avoided, particularly in curved segments, and torque alarms should prompt immediate interruption, irrigation, recapitulation, and reassessment rather than forced apical progression. Lateral brushing should be used only to relocate or relieve coronal interferences, because its application in curved regions increases wall contact and heat, reducing safety and efficiency.

Friction, lubrication, and temperature strongly influence both cutting efficiency and instrument fatigue. Maintaining continuous irrigant flow and using lubrication reduce torque demand and prevent instrument binding during rotation, particularly in curved canals. Heat buildup from friction or environmental sources increases stiffness, as the alloy shifts toward austenite at higher temperatures, which may compromise cutting efficiency and flexibility. Instruments should be removed promptly after exposure to strong oxidizers, rinsed, and dried before sterilization to preserve surface integrity.

These factors directly influence instrument lifespan; therefore, regular monitoring and timely retirement are essential. Instruments should be inspected under magnification before, during, and after use for unwinding, tip distortion, flute damage, or surface defects, and any instrument showing these signs should be discarded. Instruments should be treated as notch-sensitive, consumable tools, since cumulative contact with dentin reduces performance, and high mechanical demand in one canal can affect subsequent use. Conservative guidelines recommend single use in severe curvatures, double curvatures, or calcified canals, whereas limited reuse may be acceptable in wide, relatively straight canals with minimal workload. Instruments should not be soaked in NaOCl outside the canal. They should be rinsed and dried before sterilization and handled carefully to avoid metal-to-metal contact that may cause surface defects. Instruments should be retired at the first sign of distress, and reuse and sterilization cycles should be carefully tracked. Repeated thermal cycling and sterilization can subtly change transformation temperatures in some heat-treated NiTi alloys, reinforcing the need for conservative reuse practices.

Safe endodontic practice requires prompt responses to intraoperative warnings, since these signals indicate that the instrument is under stress. When an instrument buckles under compression, load should be reduced by confirming patency, refining the glide path, and shortening stroke length before further advancement. In narrow canals, an instrument with slightly greater column strength may be selected to maintain control. If the instrument binds or sudden torque spikes occur, the instrument should be withdrawn, the canal irrigated, the flutes cleaned, and access and coronal relief reassessed rather than increasing speed or torque to force progression. When an instrument repeatedly engages at the same depth, the operator should step back to improve the pathway or adjust the technique instead of continuing under the same conditions, ensuring safe advancement through the canal.

Skill and rehearsal complete the safety process. Preclinical training with the specific system in resin blocks or extracted teeth helps clinicians understand how the instrument engages, cuts, and releases dentin. At chairside, this experience should be combined with careful access preparation, precise glide-path creation, torque-controlled operation, short pecking motions, frequent withdrawal and cleaning, and conservative reuse policies. This coordinated approach allows clinicians to take full advantage of the flexibility, shaping efficiency, and canal-centering ability of NiTi instruments while keeping the risk of torsional overload and cyclic fatigue fracture low.^4,39^

### Current controversies and hot debates

Although the development of NiTi instruments has changed endodontics, the rapid expansion of instrument designs has created ongoing controversies regarding performance, safety, and clinical relevance. This diversity makes direct comparisons difficult and raises questions about how laboratory findings translate to clinical practice. These discussions can be illustrated by four key issues. The first addresses how multimethod research should evaluate instrument performance. The second questions whether mechanical tests conducted at a fixed temperature of 36°C accurately reflect clinical conditions. The third concerns the clinical and ethical risks of replica-like or counterfeit instruments. The fourth examines the purported benefits of flat-side designs. These issues highlight the gap that can exist between laboratory findings, marketing claims, and clinical practice.

### Multimethod versus simplified-method approaches in instrument testing

Mechanical testing of NiTi instruments has traditionally focused on isolated parameters, such as cyclic fatigue strength, torsional resistance, or bending stiffness.^95-97^ These tests provide important insights into specific aspects of instrument behavior, but their clinical relevance is limited because they only partially replicate the complex stresses observed *in vivo*.^4^ For example, a cyclic fatigue test in a standardized stainless-steel canal measures instrument lifespan under repetitive bending in a fixed curvature, but it does not account for simultaneous torsional stress, debris accumulation, irrigation dynamics, root canal anatomy, or operator variability. Similarly, torsional tests under ISO specifications quantify strength at lock-in points but cannot predict instrument behavior in curved canals with varying geometries.

Multimethod research has emerged to address these limitations.^59^ By combining multiple complementary tests such as design evaluation, mechanical testing, metallurgical assessment, shaping ability analysis, and finite-element analysis, researchers can provide a more complete understanding of instrument performance. Multimethod approaches that integrate qualitative and quantitative analyses allow cross-validation of results and improve the reliability of translating laboratory data into clinical recommendations.^3^ This approach also reduces the risk of misleading conclusions from single tests. For instance, flat-side instruments initially appeared superior in cutting ability, but multimethod studies revealed weaker performance in cyclic fatigue and torsional strength, providing a more balanced understanding of their properties.^35^ Without such triangulation, clinical decisions may rely on incomplete or biased data. While simplified methods remain useful for mechanistic studies or hypothesis generation, current best practices favor multimethod research as the most effective way to guide both manufacturers and clinicians, supporting informed decisions regarding NiTi instrument design and clinical use.^3,36^

### Mechanical tests conducted at a fixed temperature of 36 °C

**Figure 8:**
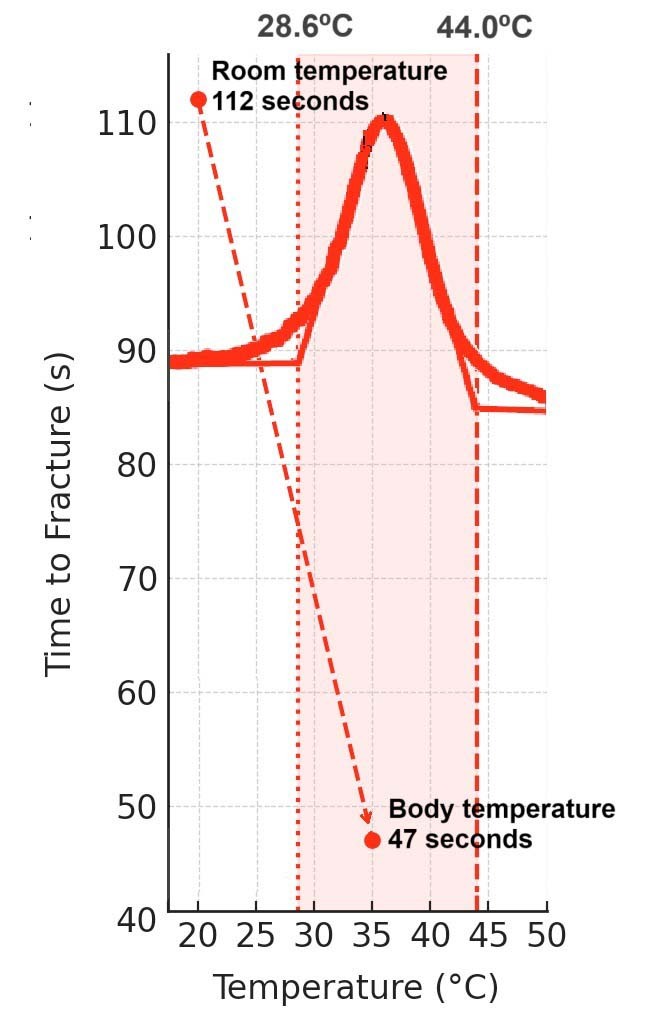
Time to fracture of ProTaper Gold F2 (Dentsply Sirona) files on cyclic fatigue testing markedly decreased (from 112 to 47 seconds) when temperature increased from 20 °C (room temperature) to 35 °C (body temperature) (Silva et al. 2025). This reduction reflects the temperature-dependent crystallographic changes of the alloy, illustrated by the red-shaded region corresponding to the onset of a phase transformation (R-phase). These findings highlight that cyclic fatigue performance is influenced not by temperature alone, but by the underlying phase transformation behavior of the alloy.

Another debated topic concerns the choice of temperature in laboratory mechanical testing. Some studies adopt 36°C as a default “body temperature,” assuming that instruments function under conditions similar to those *in vivo*.^98,99^ This assumption is problematic because intracanal temperatures are neither uniform nor constant. Recent studies indicate that temperatures during shaping range from room temperature to approximately 31–33°C, depending on irrigation flow, volume, and tooth type.^3,4,100-103^ As a result, mechanical tests conducted at a fixed 36°C may not accurately represent clinical conditions. This limitation is particularly important because NiTi alloy behavior is highly temperature-dependent (Figure 8). A heat-treated instrument with an Af temperature near 30°C behaves predominantly as martensite at 20°C but as austenite at 36°C, showing substantially different mechanical properties. Differential scanning calorimetry demonstrates this effect, showing that ProTaper Gold (Dentsply Sirona) and ProTaper Universal (Dentsply Tulsa), despite having similar geometries, have distinct transformation temperatures. Consequently, their cyclic fatigue performance differs markedly between 20°C and 35°C.^104^

This debate highlights two important methodological issues. Testing only at 36°C can overestimate instrument stiffness and underestimate fatigue resistance because canals often remain cooler during clinical use. Moreover, applying a single temperature ignores the variability among patients and clinical procedures. For instance, an irrigated canal with abundant sodium hypochlorite at room temperature may never reach 36°C. To better reflect clinical conditions, researchers increasingly recommend testing at multiple temperatures, such as 22°C and 35°C or correlating mechanical results with differential scanning calorimetry data to improve prediction of clinical performance.^3,4,102,104^

### Clinical and ethical risks of replica-like or counterfeit instruments

**Figure 9:**
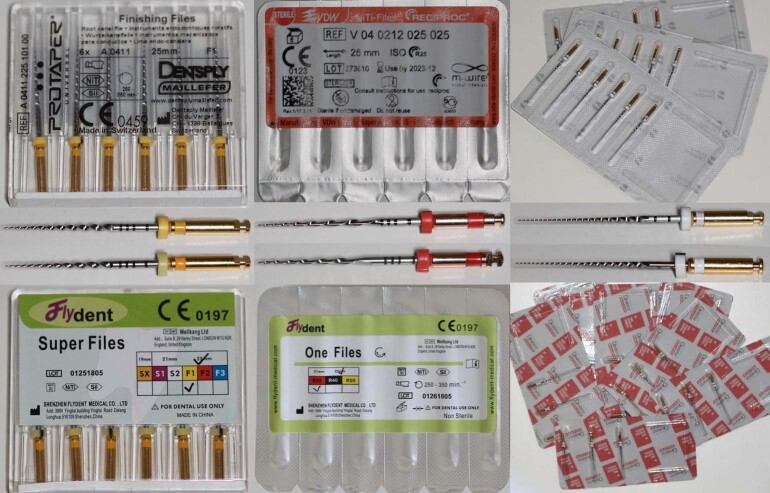
Three types of replica-like and counterfeit endodontic instruments. The top row shows the original products and the bottom row the alternative options available on the market. On the right, the ProTaper Universal F1 (Dentsply Maillefer) file with its replica-like counterpart; in the center, the Reciproc R25 (VDW) instrument with a replica-like option; and on the left, the ProGlider (Dentsply Sirona) instrument with a counterfeit file displaying the Dentsply label (this file was confirmed as counterfeit by the Dentsply company in a previous study.^23^

The global distribution of NiTi instruments has increased the availability of replica-like and counterfeit products (Figure 9), which are often sold through local distributors and online platforms. Replica-like instruments copy the sequence, nomenclature, and color coding of original systems, yet are marketed under different brand names,^106^ giving the impression of legitimacy. Counterfeit instruments, in contrast, are illegal copies that falsely claim to be genuine products,^107^ and they pose clear clinical risks. They consistently show lower cyclic fatigue resistance, higher bending stiffness, and inconsistent metallurgical properties.^107,108^ For example, counterfeit Reciproc instruments exhibited significantly shorter fatigue lifespans and altered elemental composition compared with authentic Reciproc (VDW) instruments.^107^ Similarly, a study comparing original ProTaper Next (Dentsply Sirona) instruments with replica-like and counterfeit versions found that the counterfeit instruments were the least reliable and posed the greatest safety risk.^108^ In addition to mechanical deficiencies, counterfeit products raise ethical and regulatory concerns by bypassing quality control, infringing intellectual property, and increasing the risk to patients of complications associated with instrument failure.^108^

Comparative studies show that replica-like systems, while visually similar to branded instruments and comparable in nominal composition, often differ in phase transformation temperatures, surface finish, and mechanical behavior.^107,108^. Reports also highlight quality control issues, such as fracture, unwinding, or handle loosening.^24^ Despite these concerns, their mechanical performance is inconsistent, indicating that, unlike counterfeits, replica-like instruments may not represent a major clinical risk. Given these implications, researchers and journals have stressed the need for increased vigilance and initiatives to inform practitioners.^107,108^ Despite these efforts, replica-like and counterfeit systems continue to circulate globally, representing an ongoing and unresolved challenge.

### Flat-side design: promise or pitfall?

The introduction of flat-side geometry in NiTi instruments was initially promoted as a design innovation intended to reduce metallic mass, increase flexibility, extend fatigue lifespan, and improve debris removal.^3,3,109^ Despite these claims, evidence has been inconsistent, and the benefits of flat-side instruments remain debated. An early study suggested the potential benefits of this design, reporting that F One (Fanta Dental Material Co.) files had significantly higher cyclic fatigue strength than non-flat prototypes.^33^ However, subsequent multimethod investigations challenged these results.^35,36^ Silva et al.^35^ showed that although flat-side instruments improved cutting efficiency, they performed worse in time to fracture, number of rotations to fracture, and maximum torque to fracture compared with non-flat prototypes. The study also identified manufacturing inconsistencies, such as discontinuous blades and variable S-shaped cross-sections, raising concerns about reproducibility.

Subsequent research reinforced these concerns. Comparisons of conventional, flat, and hybrid (half-flat/half-conventional) blade designs revealed that both flat and hybrid instruments had lower mechanical strength than conventional non-flat instruments.^36^ In addition, Carvalho et al.,^32^ using dynamic photoelastic analysis, found that flat-side instruments generated higher stress in curved canals, particularly in apical regions, thereby increasing the risk of distortion. Taken together, these studies suggest that the theoretical advantages of flat-side geometry are outweighed by practical limitations, including higher intracanal stress, reduced mechanical strength, and greater susceptibility to deformation. Clinicians should therefore exercise caution when considering the use of instruments with flat-side designs.

### Future perspectives

The next decade of mechanical preparation with NiTi instruments will be driven less by a single breakthrough and more by the integration of alloy science, metrology, digital planning, and data-driven clinical workflows, creating a more predictive and controlled approach to canal enlargement.

A key frontier in this evolution is imaging-guided, software-assisted planning. Cone-beam computed tomography provides detailed measurements of canal curvature, radius, width, calcifications, and access trajectory. Dedicated software can translate these measurements into individualized shaping protocols, providing clinicians with plans that specify instrument brand, kinematics, tip/taper targets, brushing or relocation points, and predicted risk zones for taper-lock, buckling, or transportation. As datasets grow, artificial intelligence may detect torque-time patterns in challenging canals and provide real-time guidance on shortening strokes, renewing the irrigant, or retiring the instrument. This technology does not replace clinical judgment; instead, it supports decision-making by aligning mechanical preparation with irrigation dynamics from the outset, reducing uncertainty and improving predictability.

Another complementary frontier is endurance-based instrument design and kinematics. Traditional fatigue analysis examines whether a component can tolerate repeated strain below a defined threshold without crack initiation, establishing a practical endurance limit. For NiTi instruments, this limit varies with phase state, temperature, surface finish, and clinical conditions, but it provides a useful framework for shaping strategy. By reducing angular excursions in reciprocation and controlling contact to keep strain at the point of maximum curvature below the threshold, an instrument may operate within a long-life regime for that canal. Although no instrument is completely unbreakable, combining low-angle reciprocation with conservative core design and optimized surfaces can create a functional endurance window, even in severely curved canals, complementing imaging-guided planning to maximize safety and efficiency.

Motors are expected to evolve from simple speed regulators into intelligent, load-aware assistants. Telemetry can record torque, angular displacement, and duty cycles, generating a live risk index that estimates the remaining useful life of the instrument during treatment. When binding or prolonged rotation occurs in a curved segment, the motor may respond automatically with micro-pauses, adjustments in angle or speed, or changes in kinematics. Integrated with imaging-derived risk maps, motors may preemptively reduce reciprocation angles within predicted high-risk zones, such as double curvatures or abrupt apical hooks, while allowing more efficient motion in safer canal segments. After the procedure, logs can be stored in a local database, refining future recommendations and supporting traceable reuse policies based on actual workload instead of arbitrary cycle counts.

Advances in materials and surface treatments are expected to complement these developments. NiTi instruments should have declared Af targets suited to their role, whether for glide path creation, shaping, or retreatment, along with documented phase behavior at 37°C. Instrument cores should be graded to limit bending strain in curves while maintaining axial support against buckling. Surfaces should be refined, combining electropolishing with controlled oxide layers or thin films, to reduce notch sensitivity without compromising cutting efficiency. These features should be validated through clinically relevant testing, including realistic temperatures, irrigants, and dynamic duty cycles rather than static bench tests, and accompanied by transparent reporting of Ms, Mf, As, Af, and finishing procedures. Although a single alloy that performs all tasks perfectly is unlikely, stress-induced martensite already provides sufficient stiffness in straight segments while allowing local compliance in curved areas. Modest segment-specific Af tuning combined with endurance-aware kinematics may further enhance performance in challenging anatomies.

Finally, clinical outcomes should guide evaluation, rather than reliance solely on mechanical measures. Key endpoints include reduced iatrogenic events, improved irrigant penetration and activation, and long-lasting restorations supported by conservative dentin preservation. Achieving these standards requires transparent reporting using standardized checklists, including phase data, surface finish, test temperature and irrigant, instrument kinematics, and imaging context. Such reporting ensures that performance claims are comparable across brands and applicable to clinical practice.

## Conclusion

The evolution of NiTi instruments has fundamentally transformed endodontic therapy, establishing them as the standard of care for root canal preparation. This review highlights that the superior performance of modern NiTi systems arises from the combined effects of advanced metallurgy, refined geometric design, and controlled kinematics.

Heat treatments, such as those producing Gold and Blue wires, increase flexibility and cyclic fatigue resistance by increasing the martensitic fraction at clinical temperatures. These advantages, however, depend on instrument design, since taper, core mass, and cross-sectional geometry control the balance between cutting efficiency, torsional strength, and canal-centering ability. NiTi instruments consistently produce more centered and anatomically respectful preparations than stainless-steel files, with continuous rotary systems often achieving the best centering. Despite these benefits, complex canal anatomy limits mechanical preparation. Both rotary and reciprocating systems leave a substantial proportion of canal walls untouched, typically 20% to 33%, highlighting that instrumentation primarily facilitates effective chemical debridement rather than providing complete cleaning alone.

Instrument failure, most commonly due to cyclic fatigue or torsional overload, remains a clinical risk that must be actively managed. Safe use requires understanding these failure mechanisms and applying conservative techniques to minimize stress. Essential strategies include establishing a reproducible glide path, using torque-controlled motors, employing short pecking motions, and adhering to strict guidelines for instrument reuse and retirement. While NiTi instrumentation improves radiographic healing compared with hand files, long-term success depends largely on biological factors such as preoperative periapical status and thorough three-dimensional disinfection. Future developments will focus on combining imaging-guided planning and artificial intelligence to create individualized, risk-assessed shaping protocols, thereby bridging laboratory data and clinical practice.

In conclusion, modern endodontic practice requires moving beyond simple comparisons of instrument systems toward a holistic approach that integrates NiTi metallurgy, instrument design, and procedural factors. By optimizing these interactions, clinicians can maximize safety and efficacy, laying the groundwork for more predictable root canal preparation.

## Data Availability

The authors declare that all data generated or analyzed during this study are included in this published article.
